# Inflammatory gut as a pathologic and therapeutic target in Parkinson’s disease

**DOI:** 10.1038/s41420-022-01175-2

**Published:** 2022-09-24

**Authors:** Jea-Young Lee, Zhen-Jie Wang, Alexa Moscatello, Chase Kingsbury, Blaise Cozene, Jeffrey Farooq, Madeline Saft, Nadia Sadanandan, Bella Gonzales-Portillo, Henry Zhang, Felipe Esparza Salazar, Alma Rosa Lezama Toledo, Germán Rivera Monroy, Reed Berlet, Cyndy D. Sanberg, Paul R. Sanberg, Cesario V. Borlongan

**Affiliations:** 1 Center of Excellence for Aging and Brain Repair, Department of Neurosurgery and Brain Repair, University of South Florida Morsani College of Medicine, Tampa, FL 33612 USA; 2 Tulane University, New Orleans, LA 70118 USA; 3 University of Michigan, Ann Arbor, MI 48109 USA; 4 Georgetown University, Washington, DC 20057 USA; 5 Northwestern University, Evanston, IL 60208 USA; 6 University of Florida, Gainesville, FL 32611 USA; 7 Centro de Investigación en Ciencias de la Salud (CICSA); FCS, Universidad Anáhuac México Campus Norte; Huixquilucan, Edo. de México, México; 8 Chicago Medical School, North Chicago, IL 60064 USA; 9 Saneron Therapeutics, Tampa, FL 33612 USA

**Keywords:** Cell death in the nervous system, Parkinson's disease

## Abstract

Parkinson’s disease (PD) remains a significant unmet clinical need. Gut dysbiosis stands as a PD pathologic source and therapeutic target. Here, we assessed the role of the gut-brain axis in PD pathology and treatment. Adult transgenic (Tg) α-synuclein-overexpressing mice served as subjects and were randomly assigned to either transplantation of vehicle or human umbilical cord blood-derived stem cells and plasma. Behavioral and immunohistochemical assays evaluated the functional outcomes following transplantation. Tg mice displayed typical motor and gut motility deficits, elevated α-synuclein levels, and dopaminergic depletion, accompanied by gut dysbiosis characterized by upregulation of microbiota and cytokines associated with inflammation in the gut and the brain. In contrast, transplanted Tg mice displayed amelioration of motor deficits, improved sparing of nigral dopaminergic neurons, and downregulation of α-synuclein and inflammatory-relevant microbiota and cytokines in both gut and brain. Parallel in vitro studies revealed that cultured dopaminergic SH-SY5Y cells exposed to homogenates of Tg mouse-derived dysbiotic gut exhibited significantly reduced cell viability and elevated inflammatory signals compared to wild-type mouse-derived gut homogenates. Moreover, treatment with human umbilical cord blood-derived stem cells and plasma improved cell viability and decreased inflammation in dysbiotic gut-exposed SH-SY5Y cells. Intravenous transplantation of human umbilical cord blood-derived stem/progenitor cells and plasma reduced inflammatory microbiota and cytokine, and dampened α-synuclein overload in the gut and the brain of adult α-synuclein-overexpressing Tg mice. Our findings advance the gut-brain axis as a key pathological origin, as well as a robust therapeutic target for PD.

## Abstract (web summary)

*Gut-Brain Axis as a PD Pathologic Source and Therapeutic Target*. The PD murine model of α-synuclein overexpression at around 8 weeks of age manifests gut dysbiosis, characterized by inflammation-specific microbiota and cytokines, which can trigger brain neurodegeneration, especially dopaminergic depletion reminiscent of PD pathology. Targeting the dysbiotic gut via intravenous hUCB stem cell transplantation can render gut homeostasis and sequester peripheral as well as central inflammation, leading to brain repair and amelioration of PD behavioral and histological deficits.

## Graphical abstract

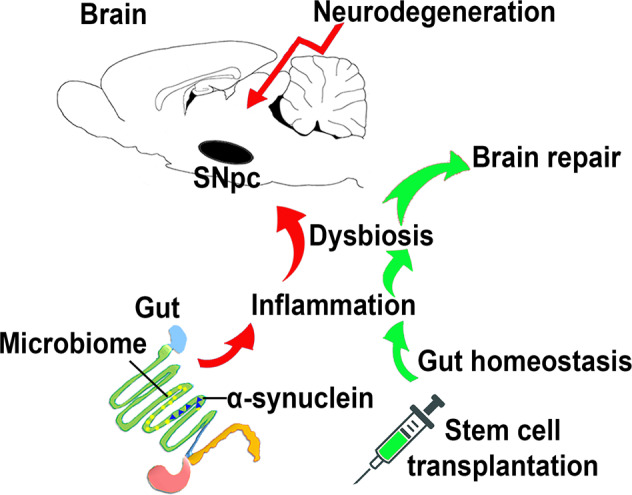

## Introduction

Parkinson's disease (PD) is the second most prevalent neurodegenerative disorder affecting aging people [1]. PD is characterized by the loss and damage of dopaminergic neurons located in the substantia nigra pars compacta (SNpc), which leads to a deficiency of dopamine (DA) in the striatum and the formation of Lewy bodies containing aggregates of α-synuclein [1, 2]. PD presents with progressive motor abnormalities such as muscle stiffness, tremors, impaired balance, bradykinesia, and gait freezing [3]. However, it also presents non-motor symptoms, especially cognitive deterioration and abnormalities in intestinal function, which favors the disease’s morbidity [3, 4]. Despite scientific advances regarding PD, there is no cure, and only palliative treatments are available to attenuate the disease motor and non-motor symptoms. Therapeutic approaches focus on pharmacologastrocal replacement of striatal dopamine, with levodopa as the gold standard pharmacological treatment [1, 5].

Stem cell-based regenerative medicine has been introduced to exogenously replace dopamine and stimulate endogenous brain repair [6, 7]. Cell therapy stands as a disease-modifying strategy for PD [8, 9]. Dopaminergic cell replacement using fetal derived dopaminergic cells into PD patients revealed survival of the transplant, as well as integration of the grafted cells into the host dopaminergic network accompanied by modest clinical outcomes [10]. However, some patients who received fetal dopaminergic transplants also exhibited worsening dyskensias [11]. In tandem with dopaminergic cell replacement, the transplantation of stem cells may also confer by-stander effects via secretion of therapeutic substance [12], in part acting through the gut microbiome to reduce inflammation [1]. That the gut-brain axis (GBA) is targeted by transplanted stem cells offers a new avenue in rescuing dopaminergic cell loss and ameliorating parkinsonian symptoms.

Human umbilical cord blood (hUCB) stands as potent stem cell donor source for transplantation in neurodegenerative diseases, including PD [1, 13–15]. Behavioral and histological analyses, with emphasis on gut microbiota and its inflammatory profile revealed that clinically relevant animal models of neurological disorders when transplanted with stem cells exhibit functional improvements [1, 13–15]. These encouraging results implicating the role of gut microbiota and inflammation in disease pathology and as a target for therapeutic development have been observed in our PD models of 6-hydroxydopamine (6-OHDA) and 1-methyl-4-phenyl-1,2,3,6-tetrahydro-pyridine (MPTP) [1, 15], suggesting that gut-based stem cell therapy could be used as stand-alone or an adjuvant treatment for PD, limiting and reducing the effects of dopaminergic cell loss [1, 15, 16]. Lewy bodies (LB) have been recognized as the main pathological hallmark of some neurodegenerative diseases such as PD [17]. Due to the overexpression of α-synuclein in LB present in many familial forms of PD [18], α-synuclein overexpressing transgenic (Tg) mice becomes a viable model to assess PD pathology and treatment with hUCB stem cell therapy. The neurotoxin models of 6-OHDA and MPTP, which create abrupt near complete depletion of nigrostriatal dopamine, replicate the chronic stage of PD, whereas the α-synuclein overexpressing Tg mice mimics a slowly progressive dopaminergic degeneration. Rigorous examination of PD pathology and novel therapeutics in both acute and chronic stages of the disease will enhance the successful translation of laboratory findings from the laboratory to the clinic [19]. The present study evaluated the α-synuclein overexpression Tg murine model of PD to further explore the brain-gut hypothesis compared to our previous models using MPTP and 6-OHDA insults [1, 15]. In particular, we investigated gut dysbiosis at the early progression of PD pathology and assessed the therapeutic potential of intravenous hUCB-derived plasma (P) and a combination of hUCB stem cells with hUCB-derived plasma (hUCB+P). We hypothesized that gut dysbiosis, likely acting through inflammation-relevant microbiota and cytokines, predisposed nigrostriatal dopaminergic depletion. Furthermore, we advanced the premise that P and hUCB+P treatment, when initiated at the early phase of disease progression in Tg mice (as opposed to the late stage in our previous neurotoxin models [1, 15]), would sequester gut inflammation and dopaminergic neurodegeneration. PD-sensitive motor and non-motor symptoms, coupled with in vivo and in vitro GBA analyses of inflammation and neurotoxicity, revealed the pathological link between gut dysbiosis and brain dopaminergic neurodegeneration. Abrogating the dysbiotic gut via stem cell therapy may confer a disease-modifying treatment for PD.

## Results

### Behavioral analyses

**Fig. 1:**
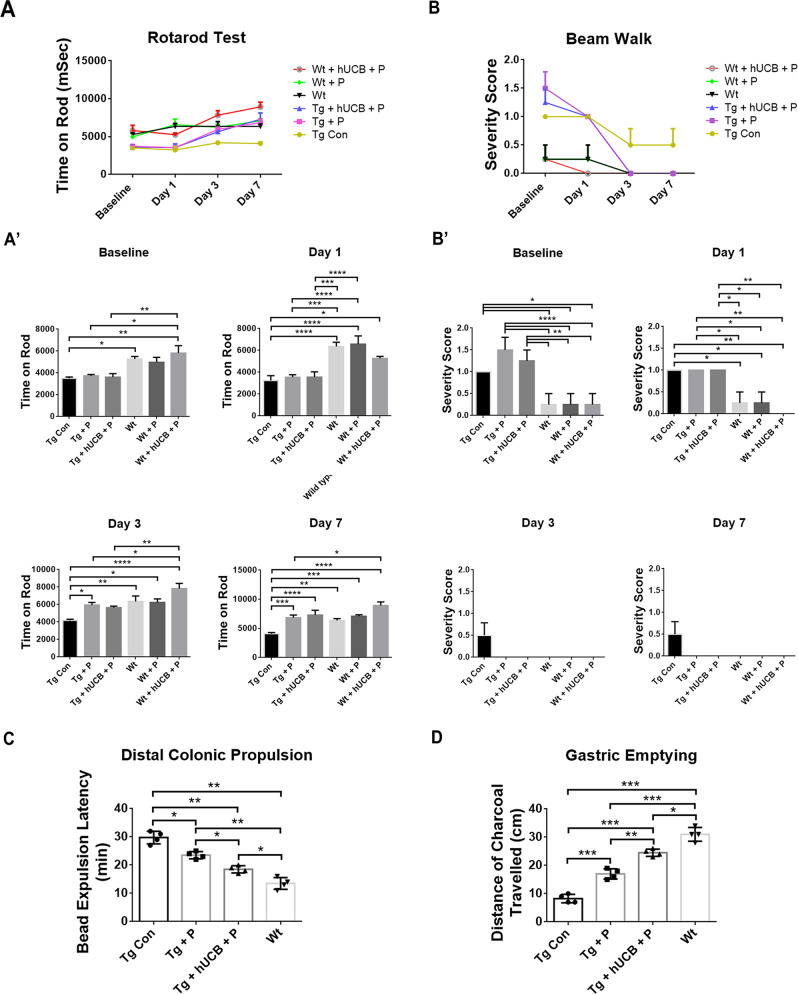
Behavioral test and gut functional assays. **A** The Rotarod test demonstrated significant differences (****p < 0.0001) in time latency to fall between treated and control groups of Tg animals at day 7. **A’** During each timepoint, performance on the Rotarod test was measured for significance for each group, with P and hUCB+P treatment significantly mitigating (*p < 0.05) motor coordination and balance deficits in Tg compared to Wt (*p < 0.05, **p < 0.01, ***p < 0.001, ****p < 0.0001). **B** The beam walk test revealed significant reductions (****p < 0.0001) in severity scores of Tg between baseline and day 7. **B’** P and hUCB+P treatment significantly reduced (*p < 0.05) locomotor deficits in Tg compared to Wt at baseline and day 1 (*p < 0.05, **p < 0.01, ****p < 0.0001). **C** Colonic motility showed that Wt had a short latency for bead expulsion, whereas Tg displayed a significantly much longer time in bead expulsion (**p < 0.01). Treatment with P and hUCB+P treatment improved colonic motility in Tg but these treated Tg mice remained functionally deficient compared to Wt (*p < 0.05, **p < 0.01). **D** Gastric emptying showed that Wt had the longest intestinal distance of charcoal traveled, whereas Tg had the shortest length (*p < 0.05), (**p < 0.01, ***p < 0.001). Treatment with P and hUCB+P treatment significantly increased the intestinal length traveled by the charcoal in Tg but these treated Tg mice remained deficient in gastric emptying compared to Wt (*p < 0.05), **p < 0.01, ***p < 0.001.

Rotarod behavioral testing was conducted to assess the differences in motor coordination and balance between Tg and Wt animals (Fig. 1A). Rotarod behavioral testing demonstrated significant differences in treatment effects of Tg animals at day 7 (*p* < 0.0001; F_5, 15_ = 11.45), with P and hUCB+P treatment partially mitigating motor coordination and balance deficits present in Tg animals compared to Wt animals (*p* < 0.05) (Fig. 1A’). Locomotor performance was assessed using the beam walk test (Fig. 1B). Beam walk behavioral testing demonstrated significant differences in treatment vs time effects of Tg animals at day 7 (*p* = 0.0004; F_15, 72_ = 3.280), with P and hUCB+P treatment partially mitigating locomotor deficits present in Tg animals compared to Wt animals (*p* < 0.05) (Fig. 1B’).

### Gut functional assays

We evaluated the functional dysfunction of the gastrointestinal (GI) system via colonic motility and GI transit tests. Colonic motility (Fig. 1C) revealed that Wt mice exhibited the most efficient colonic motility as evidenced by a short latency for bead expulsion, whereas Tg mice displayed a significantly much longer time in bead expulsion (*p*’s < 0.05). Treatment with P and hUCB+P treatment improved colonic motility in Tg animals but these treated Tg animals remained functionally deficient compared to Wt animals (*p*’s < 0.05) (Fig. 1C). Similarly, gastric emptying (Fig. 1D) revealed that Wt mice exhibited efficient gastric emptying with the charcoal traveling the longest in these animals (~30 cm), whereas Tg mice showed the shortest intestinal length (~8 cm) (*p*’s *<* 0.05). Treatment with P and hUCB+P treatment significantly increased the intestinal length traveled by the charcoal in Tg animals (~17–23 cm) but these treated Tg animals still displayed dysfunctional gastric emptying compared to Wt animals (*p*’s < 0.05) (Fig. 1D).

### Gut microbiome analysis

**Fig. 2:**
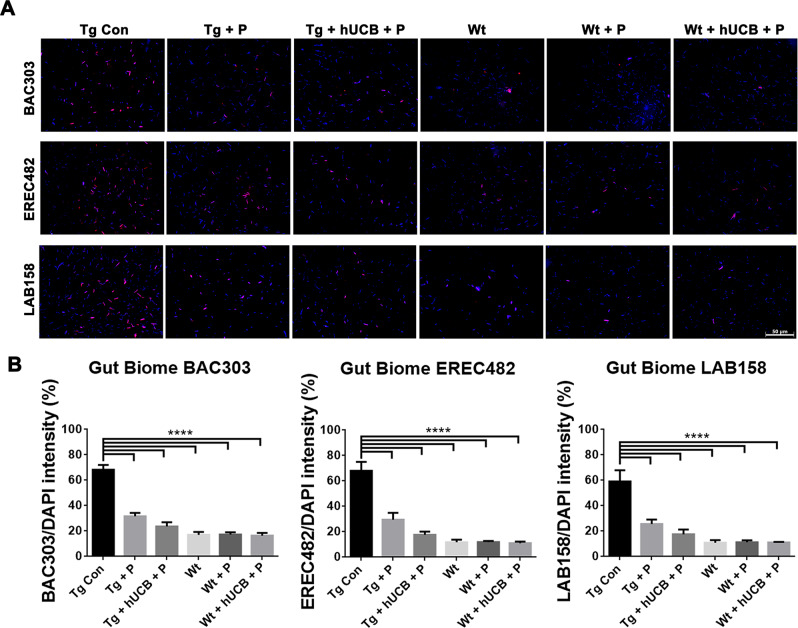
Gut microbiome differences in PD mouse model. **A** FISH analysis was used to distinguish the specific microbiota within the feces of the animals using probes for BAC303 (red), EREC482 (red), and LAB158 (red) with DAPI (blue). Immunofluorescent microscopy was used to collect the images of representative samples from each animal group (Scale bar = 50 um). **B** Differences in gut biome was measured for significance for each group by calculating probe fluorescence intensity/DAPI. ANOVA revealed significantly elevated BAC303 levels (****p < 0.0001), EREC482 levels (****p < 0.0001), and LAB158 levels (****p < 0.0001) in Tg control groups compared to both treatment groups and Wt animals (****p < 0.0001).

Fluorescent in‐situ hybridization (FISH) analysis was used to distinguish the specific microbiota within the feces of the animals in this study using DAPI staining and probes for BAC303, EREC482, and LAB158 (Fig. 2A). Tg animals exhibited significantly elevated BAC303 levels (*p* < 0.0001; F_5, 48_ = 56.62), EREC482 levels (*p* < 0.0001; F_5, 48_ = 32.29), and LAB158 levels (*p* < 0.0001; F_5, 48_ = 18.71) compared to Wt animals (Fig. 2B), with P and hUCB+P treatment partially mitigating the number of inflammatory bacteria present in Tg animals (*p* < 0.05).

### Histopathology of PD brain

**Fig. 3:**
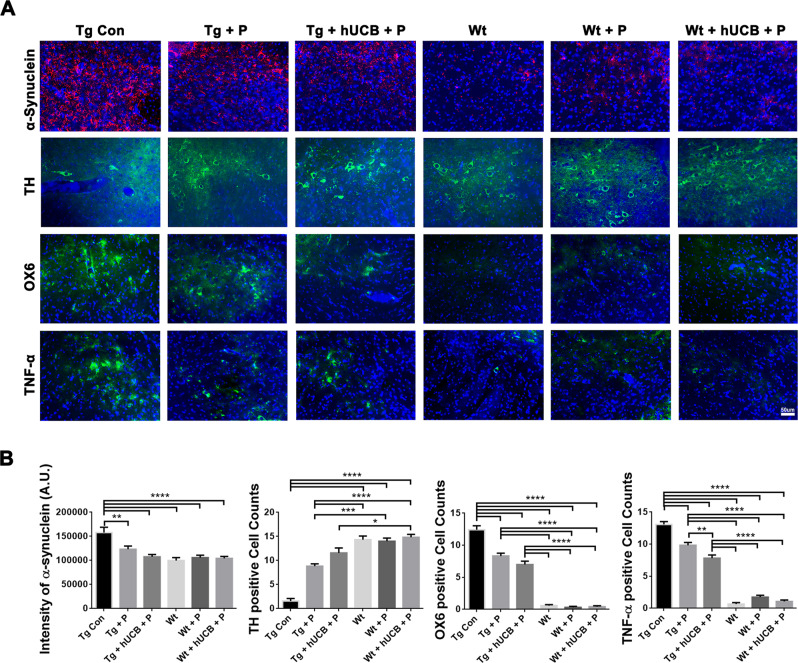
Substantia Nigra dopaminergic cell survival, inflammation, and α-synuclein load. **A** Immunohistochemical staining of the bilateral substantia nigra pars compacta (SNpc) was conducted to elucidate alterations in cell population expression for α-synuclein (red), TH (green), OX6 (green), and TNF-α (green) with DAPI (blue). Immunofluorescent microscopy was used to collect the images of representative samples from each animal group (Scale bar = 50 um). **B** ANOVA revealed significantly elevated α-synuclein intensity level (****p < 0.0001), significantly lower TH expression (****p < 0.0001), significantly elevated OX6 expression (****p < 0.0001), and significantly elevated TNF-α expression (****p < 0.0001) in Tg vs Wt animals. Additionally, post hoc Bonferroni’s test analysis revealed P and hUCB+P treatment significantly lowered (*p < 0.05) the inflammatory response in Tg animals (*p < 0.05, **p < 0.01, ***p < 0.001, ****p < 0.0001).

Image analysis of α-synuclein staining (Fig. 3A) revealed significantly elevated α-synuclein levels in Tg vs Wt animals (*p* < 0.0001; F_5, 55_ = 10.60) with P treatment and hUCB+P treatment mitigating the overexpression of α-synuclein in Tg mice (*p* < 0.05) (Fig. 3B). Image analysis with of TH staining (Fig. 3A) revealed that Tg animals showed a significantly greater (*p* < 0.0001; F_5, 55_ = 46.62) degree of dopaminergic depletion compared to Wt mice (Fig. 3B). P treatment in Tg animals moderately rescued neuron survival, while hUCB+P treatment further attenuated dopaminergic cell death (*p* < 0.05). Additionally, OX6 and TNF-α staining demonstrated analogous changes in immune cell recruitment and inflammation in the SNpc, respectively (Fig. 3A). Tg animals exhibited significantly elevated OX6 (*P* < 0.0001; F_5, 55_ = 185.2), and TNF-α (*P* < 0.0001; F_5, 55_ = 183.2), compared to Wt animals (Fig. 3B) with P and hUCB+P treatment partially mitigating the robust immune and inflammatory response in Tg animals (*p* < 0.05). In hUCB+P treated animals, graft survival was sporadic (less than 10 HuNu immunopositive cells per mouse) in the SNpc.

### Histopathology of Gut mucosa

**Fig. 4:**
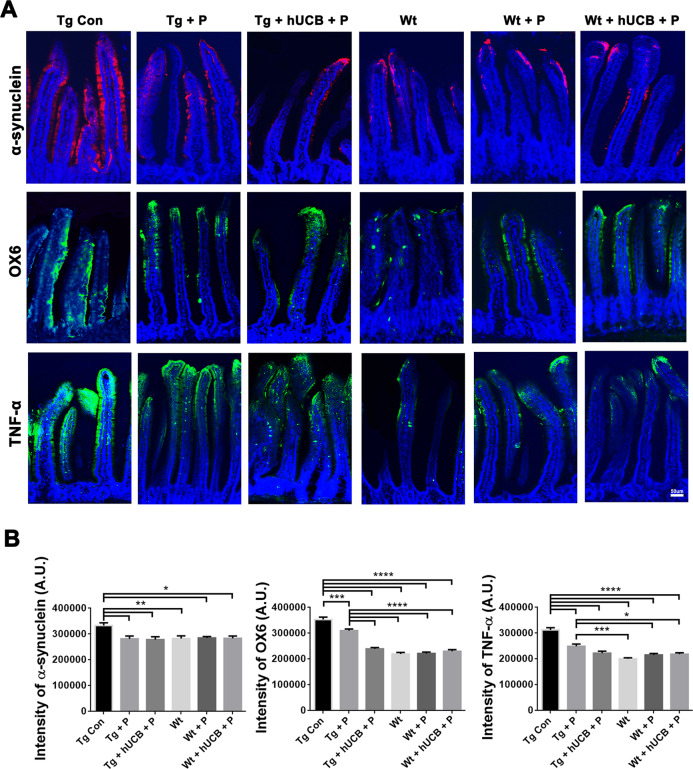
Intestinal villi inflammation, and α-synuclein load. **A** Immunohistochemical staining of the intestinal villi was conducted to elucidate alterations in cell population expression for α-synuclein (red), OX6 (green), and TNF-α (green) with DAPI (blue). Immunofluorescent microscopy was used to collect the images, of which representative samples are displayed according to animal group and factor of interest (Scale bar = 50 um). **B** ANOVA revealed significantly elevated α-synuclein intensity level (*p < 0.01) significantly elevated OX6 expression (****p < 0.0001), and significantly elevated TNF-α expression (****p < 0.0001) in Tg vs Wt animals. Additionally, post hoc Bonferroni’s test analysis revealed P and hUCB+P treatment significantly reduced (*p < 0.05) the inflammatory response in Tg animals (*p < 0.05, **p < 0.01, ***p < 0.001, ****p < 0.0001).

Immunostaining with α-synuclein (Fig. 4A) identified a significantly altered protein expression between Tg and Wt animals (Fig. 4B) in the gut mucosa (*p* < 0.01; F_5, 40_ = 4.498) with P and hUCB+P treatment partially reducing the expression of α-synuclein in Tg animals (*p* < 0.05). Immunostaining of inflammatory markers OX6 and TNF-α identified significant differences in immune cell recruitment and inflammatory responses in intestinal villi between Tg and Wt animals (Fig. 4A). Tg animals exhibited significantly elevated OX6 (*p* < 0.0001; F_5, 55_ = 68.77) and TNF-α (*p* < 0.0001; F_5, 55_ = 35.78) expression compared to Wt animals (Fig. 4B), with P and hUCB+P treatment partially suppressing the elevated immune and inflammatory response in Tg animals (*p* < 0.05). In hUCB+P treated animals, HuNu immunostaining revealed modest graft survival (about 400–800 immunopositive cells per mouse) in the gut mucosa.

### Gut homogenates and cultured dopaminergic cell assays

ANOVA revealed significant treatment effects of Wt and Tg gut exposure on dopaminergic SH-SY5Y cells (F_3,140_ = 49.32, *p* < 0.0001) (Fig. 1). Cultured SH-SY5Y cells under Wt gut homogenates displayed robust cell viability with trace levels of TNF-α comparable to ambient cell culture condition. On the other hand, exposure of SH-SY5Y cells to Tg gut revealed about 25% reduction in cell viability accompanied by about 10-fold upregulation in TNF-α levels compared to Wt-exposed SH-SY5Y cells (*p*’s < 0.05). Interestingly, although exposure of SH-SY5Y cells to Tg gut+P or Tg gut+hUCB+P still resulted in significantly lower cell viability and higher TNF-α expression compared to Wt gut-exposed SH-SY5Y cells (*p*’s < 0.05), these treatments increased cell viability by 13–20% and decreased TNF-α by about 27–68% compared to Tg gut-exposed SH-SY5Y cells (*p*’s < 0.05). The Tg gut+hUCB+P led to significantly improved cell viability and reduced TNF-α expression compared to Tg gut+P (*p*’s < 0.05).

**Fig. 5:**
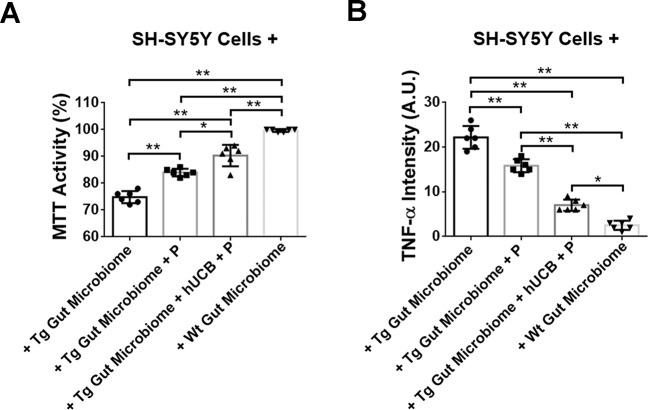
Probing the gut-brain axis via cell culture mechanistic paradigm. MTT activity assay revealed cultured SH-SY5Y cells under Wt gut homogenates displayed robust cell viability (**A**) with only trace levels of TNF-α (**B**). In contrast, exposure of SH-SY5Y cells to Tg gut reduced cell viability by about 25% and increased by about 10-fold TNF-α levels compared to Wt-exposed SH-SY5Y cells (***p < 0.05, ****p < 0.01). Tg gut+P or Tg gut+hUCB+P still also showed lower cell viability and higher TNF-α expression compared to Wt gut-exposed SH-SY5Y cells (***p < 0.05, ****p < 0.01), but they increased cell viability by about 13–20% and decreased TNF-α by about 27–68% compared to Tg gut-exposed SH-SY5Y cells (***p < 0.05, ****p < 0.01), with the Tg gut+hUCB+P more effectively sequestering neurodegeneration and inflammation than Tg gut+P (***p < 0.05, ****p < 0.01).

## Discussion

The present study provided evidence implicating the critical role of the GBA in the pathology and treatment of PD using the α-synuclein-overexpressing Tg mice. Tg mice displayed motor and non-motor (GI-related) symptoms that were quantitatively discernable from Wt mice. Both gut and brain from Tg mice exhibited upregulation of inflammation-associated microbiota and cytokines that closely approximated the α-synuclein-plagued neurodegeneration. Intravenous transplantation of P and hUCB+P partially improved motor and non-motor symptoms compared to Tg control mice. Furthermore, transplanted Tg mice displayed significantly reduced inflammation-linked gut microbiota, decreased pro-inflammatory cytokine concentrations, and reduced gut and brain α-synuclein. Probing this GBA in vitro revealed that feeding cultured dopaminergic cells directly with dysbiotic gut homogenates from Tg mice recapitulated the neurodegenerative and inflammatory microenvironment of PD. The dysbiotic gut in Tg mice with its signature neurotoxic α-synuclein was associated with deleterious inflammatory microbiota and cytokines that manifested in the brain, likely contributing to the neurodegenerative dopaminergic cell loss reminiscent of PD. Abrogating this dysfunctional GBA may prove clinically relevant for developing novel PD treatments.

Stem cell therapy exerts neuroprotection in multiple models of PD, such as MPTP [20–26], 6-OHDA [27], and α-synuclein [28]. Transplantation of hUCB-derived stem cells alleviates PD manifestations, such as motor deficits [20, 26], heightened oxidative stress and apoptosis [20, 21], and rampant inflammation centrally and peripherally [21]. Aberrant gut dysbiosis and intestinal inflammation precede PD pathology [29–32]. Interestingly, α-synuclein mice exhibited exacerbated motor deficits after receiving gut microbiota from PD patients [33], suggesting that gut dysbiosis represents a disease biomarker [33, 34] as well as a therapeutic target [35].

We previously reported GBA dysbiosis in MPTP and 6-OHDA animals, characterized by motor function and gut motility deficits and upregulated inflammation cytokines in both the gut and brain accompanying the dopaminergic cells in the SNpc [1, 15]. Specific inflammation-associated microbiomes, namely BAC303, EREC482, and LAB158, were detected in the gut of these neurotoxin-lesioned PD animals [1, 15]. Intravenous transplantation with P and hUCB+P reduced these inflammatory microbiota in the gut, as well as the inflammatory cytokines in both gut and brain that possibly mediated the improvement in motor and non-motor function and the survival of dopaminergic cells in the SNpc [1, 15]. Because both neurotoxin models of PD are considered chronic, with MPTP and 6-OHDA causing abrupt and near complete depletion of dopaminergic neurons within days post-neurotoxin infusion, a more clinically relevant animals that mimics the slow, progressive neurodegeneration may provide a better platform to examine the GBA contribution to the PD pathology and therapy. Our present study employed the α-synuclein overexpressing Tg mice, specifically monitoring the GBA perturbations around 14 months of age when PD symptoms manifest in these animals. Reminiscent of the neurotoxin models, Tg mice exhibited the inflammation-plagued microbiome that accompanied gut and brain inflammation and neurodegeneration, and motor and GI deficits, which were reversed by intravenous P and hUCB+P transplantation. These promising data in all three of PD models support the active participation of the GBA in the early and late stages of neurodegeneration and also advance the potential of cell-based regenerative medicine for PD.

That the GBA closely participates to PD neurodegeneration and inflammatory response was captured in the present in vitro mechanistic probing experiment. Here, we showed that the Wt gut appears conducive for the growth of dopaminergic cells as this condition produced robust cell viability and minimal inflammatory response similar to standard cell culture medium. The significant reduction in cell viability coupled with massive upregulation of inflammation following Tg gut exposure of the dopaminergic cells demonstrates direct evidence that the dysbiotic gut confers neurodegenerative and inflammatory signals that are deleterious to cell survival. Since we detected upregulation of three inflammation-associated microbiota (i.e., BAC303, EREC482, and LAB158) in the Tg gut, it is most likely that these specific microbiomes actively modulated the observed neurodegeneration and inflammation. Interestingly, this GBA in vitro paradigm also offered a glimpse on therapeutically altering the dysbiotic gut to sequester cell death. Treatment of the Tg gut homogenates with P or hUCB+P proved beneficial in improving survival and reducing inflammation in dopaminergic cells.

The reduction in α-synuclein suggests protein clearance as a mechanism of action in this stem cell neuroprotection. The reticuloendothelial system (RES) is composed of tissue resident macrophages and blood-borne monocytes, and may act either through phagocytic or pinocytic process, in removing particulates from the lymph and blood [36]. Traditionally, tissue components of RES include spleen, liver, lymph nodes, bone marrow, and lung [37], with the gut as a recent addition [38]. Such clearance of particulates may correspond to an immunomodulatory action of RES, in particular the gut [39], highlighting its crucial role in neuroprotection. To this end, whereas the conventional concept of PD entails a neuron-mediated α-synuclein neurodegeneration, an endothelial-associated vascular signaling pathway may contribute to the disease pathology. Of note, while macrophages and monocytes have been traditionally thought to comprise RES, the endothelial cells have emerged as the major cell type of this system [38, 39]. Moreover, endothelial cells are distributed in spleen [39] and gut [38], and may commit to a phagocytic phenotype in response to injury [40], suggesting the close involvement of the vascular signaling pathway in neurodegeneration. This vascular network of RES may facilitate the GBA crosstalk, allowing the flow of its mediators, such as gut microbes, inflammatory cytokines, and immunomodulatory cells, to shuttle across the blood brain barrier in dampening gut and brain α-synuclein overload.

Some limitations with this study are associated with the technique used to assess gut microbiomes. Proteomic, lipidomic, and metabolomics assessment of the microbiome may reveal novel cell death and survival signaling pathways. Recognizing that the microbiome is subject to the microenvironment and housing, animal diet and germ-free caging conditions should be elucidated to uncover any correlation between the environmental effects and the resulting microbiome at pre- and post-PD induction and stem cell transplantation. In-depth analysis of the gut homogenate content and upregulating or blocking the microbiomes, such as the three microbiota we identified here, may also prove meritorious in further demonstrating the cause-and-effect interaction between the gut and the brain. Key biological variables must be considered such as animal age and gender (i.e., only male mice were used here), and the incorporation of relevant PD risk or co-morbid factors [41, 42] to closely recapitulate the clinical setting of the disease. Moreover, the present short-term study warrants a follow-up long-term investigation in view of PD as a chronic neurodegenerative disorder. Although the robust functional outcomes were observed, monitoring of long-lasting effects of the transplant treatments is required in order to unequivocally conclude that our proposed stem cell therapy promotes a stable disease-modifying treatment for PD. Translational studies to optimize the timing, dose, and delivery of plasma and stem cells may enhance the safety and efficacy of cell therapy for clinical translation [1, 15, 43, 44]. Finally, we need to adhere to a cautious approach in advancing the causality role of microbiota in human disease especially when employing rodent models because of distinct species microbiome profiles [45].

Aberrant perturbations in inflammatory microbiomes in the GBA may predispose PD. Similar to MPTP and 6-OHDA animals [1, 15], α-synuclein-overexpressing Tg mice exhibited a signature profile of inflammatory gut microbiomes that manifest into peripheral and central inflammatory signals culminating into neurodegeneration with phenotypic motor and non-motor GI symptoms. Intravenous cell-based treatments in thse Tg mice led to reductions in inflammatory microbiota, cytokines, and α-synuclein in the gut and in the brain coincident with improved motor function and less dopaminergic cell loss in SNpc. Targeting the gut microbiome [46] via stem cell-based regenerative medicine sequesters the destructive pro-inflammation associated with the neurodegenerative pathology of PD.

## Method

### Human umbilical cord blood cells and plasma

Saneron CCEL Therapeutics, Inc. supplied the frozen hUCB cells and plasma. The Sepax 2 full-automated cell processing system (Biosafe America Inc., Houston, TX) separated the hUCB cells and plasma for use. Before freezing, the BacT/ALERT Microbial detection system (bioMérieux, Durham, NC) was used to examine the hUCB cells and plasma units as aseptic. The hUCB units were purchased commercially from GenCure (TCBB, West San Antonio, Texas) by Saneron CCELTherapeutics, Inc. for research purposes under USF IRB# 131111. The de-identified hUCB units were processed by Saneron CCEL Therapeutics, Inc. using the Sepax 2 full-automated cell processing system (Biosafe America Inc., Houston, TX) which allowed for the sterile collection of both hUCB cells and plasma. Results for infectious disease testing of maternal blood samples, collected shortly after birth, was provided by GenCure, for infectious disease markers of HIV, hepatitis B and C, syphilis, CMV, and HTLV I&II. Each hUCB unit in the study was negative for all infectious disease markers. Saneron CCEL Therapeutics, Inc. supplied all the processed, cryopreserved, hUCB cells and plasma that was used in this study.

### Animal preparation and transplantation

**Fig. 6:**
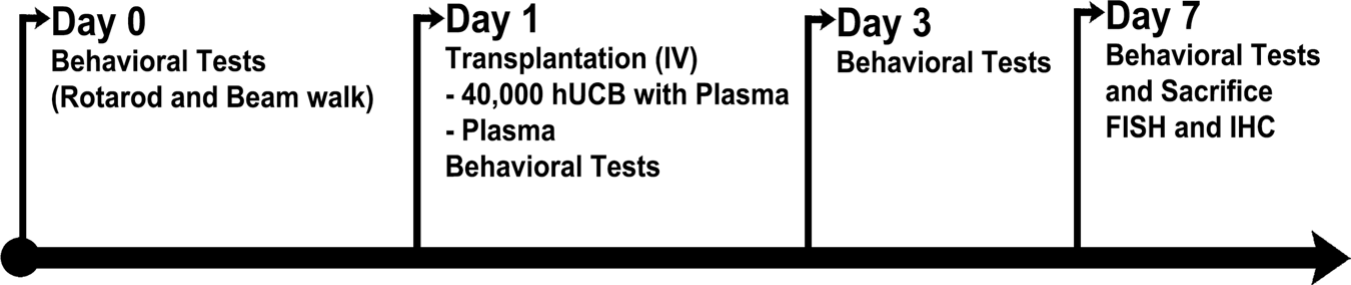
Treatment timeline. Behavioral tests at baseline were performed on Day 0 prior to transplantation. On Day 1, intravenous transplantation with P or hUCB+P was performed. Behavioral tests were conducted again on day 1, 3, and 7 post transplantation. Animals were sacrificed on day 7 for brain and gut for fluorescent in‐situ hybridization (FISH) and immunohistochemistry to assess PD histopathology.

All experimental procedures were approved by the University of South Florida Institutional Animal Care and Use Committee (IACUC) and followed the ARRIVE 2.0 guidelines [47]. All investigators were blind to the treatment condition until after completion of all data analyses. This study used 14 months-old male C57BL/6NJ (wild type or Wt) and C57BL/6N-Tg (Thy1-SNCA)15Mjff/J mice (transgenic or Tg) (The Jackson Laboratory, Bar Harbor, Me). The Tg mice overexpressed the wild type human α-synuclein, with validated phenotypic PD-like progressive nigrostriatal dopamine depletion and motor deficits [48]. All mice were included in the study, had free access to food and water, and housed under normal conditions (20 °C, 50% relative humidity, and a 12-h light/dark cycle). Mice were randomly assigned by a staff not involved in the study to one group: Wt Con (*n* = 8), Wt + P (*n* = 8), Wt + hUCB+P (*n* = 8), Tg Con (*n* = 7), Tg + P (*n* = 7) and Tg+hUCB+P (*n* = 7). 0.4 × 10^6^ hUCB cells in 50 μL of hUCB plasma or only 50 μL of hUCB plasma were intravenously injected using the jugular vein to the treatment group (Fig. 6). Sample size ensured adequate power to detect 25% treatment effect size.

### Behavioral tests (Rotarod and Beam Walk)

The Rotarod test was used to evaluate motor functional change. Briefly, mice were forced to run on a rotating drum (IITC Life Science) with speeds starting at 4 rpm and accelerating to 40 rpm in 300 s. Three consecutive trials were conducted for each mouse with an interval of 15 min. The time at which a mouse fell off the drum was recorded as the latency to fall. The beam walk test was performed to assess forelimb and hindlimb function. To assess beam walking ability, examiners graded 0 for rats that readily traversed a 2.4-cm-wide, 80-cm-long beam to 3 for rats unable to stay on the beam for 10 s.

### Distal colonic propulsion

A 0.5-mm-diameter glass bead was placed approximately 2 cm from each rat’s anus into the distal colon on the last survival day to evaluate colonic propulsion. Each animal was then placed back in their cages without food or water following insertion of the glass bead. The animals were closely monitored to observe any abnormal changes in behavior. The time of glass bead excretion, called mean expulsion time, was recorded for each individual animal to the nearest 1.0 s.

### Gastric emptying

On the last survival day, a solution of charcoal (10%) and acacia gum (2%) were administered via oral gavage the animals. The animals were then placed back in their cages for 30 min prior to euthanizing by carbon dioxide. Each animal’s intestine was harvested, and the length (cm) that the charcoal meal moved was recorded.

### Tissue collection

Under deep anesthesia, the mice were sacrificed at 7 days. Animals were perfused transcardially with approximately 60 mL of ice-cold PBS followed by 60 mL of 4% paraformaldehyde in PBS. The intestines and brains of these animals were removed and post‐fixed in the same fixative for 24 h at 4 °C. These tissues were then transferred to 30% sucrose in PB for at least 16 h prior to tissue sectioning. Coronal sections were cut (40 µm) using a cryostat, then collected and stored in a cryoprotectant solution at −20 °C.

### Microbiome analysis

The gut microbiome was examined using the following measures to determine the prevalence of particular inflammatory, possibly harmful, bacterial species. The mice’s fecal microbiota was analyzed and identified using fluorescent in-situ hybridization (FISH). 1 g of collected feces was suspended in PBS (2 mL). Repetitive pipetting was performed to homogenize the fecal matter, followed by mixing with a vortex and addition of 6 mL of 4% paraformaldehyde for fixation. Following an overnight incubation period at 4 °C, the fecal tubes were centrifuged for 5 min at 10 g to sediment the undigested matter. The supernatant was placed in a new tube and centrifuged an additional time. The supernatant was then transferred to a new tube, and centrifugation was repeated for 5 min at 60 g. The supernatant was removed, and the resulting bacterial pellet was suspended in 500 μL of hybridization solution including 50% (v/v) formamide, 100 μg/mL salmon sperm DNA, 5x saline-sodium citrate buffer, and 0.1% (v/v) Tween 20. The samples were incubated for 30 min 37 °C. 50 μL of each sample were then added into a new tube with 2.7 μL of fluorescently labeled oligonucleotides. Following an incubation period at 45 °C for 2.5 h in the dark, the tubes were centrifuged at 60 g for 5 min. The supernatant was removed and discarded, and the pellet was resuspended in 20 μL of 0.1 M sterile sodium chloride. This washing was repeated 2 additional times, and the pellets were then resuspended in 20 μL of PBS. The samples were centrifuged at 13,000 g for 5 min. 10 μL was transferred to a new slide sterilized with EtOH. 5 μL of Vectashield mounting medium with DAPI was added to the slides and cover-slipped. An Olympus FV1000 laser scanning confocal microscope with Fluoview SV1000 imaging software was used to collect the images at 40x.

### Immunofluorescence

Staining for tyrosine hydroxylase (TH) and α-synuclein was conducted on every sixth coronal section throughout the entire SNpc or gut. In all animals, sections were anatomically matched. Eight coronal sections from each mouse were washed three times in 0.1 M PBS. Afterwards, all slide washed with PBS three times at 10 min each. Next, all sections were incubated for 1 h in a blocking solution composed PBS supplemented with 3% normal goat serum and 0.2% Triton X‐100. Sections were then incubated overnight at 4 °C with rabbit polyclonal TH (1:100 TH, AB152; Millipore, Burlington, MA) or rabbit polyclonal α-synuclein (1:250, NBP2-15365; NOVUS, Centennial, CO) antibody diluted in PBS supplemented with 3% normal goat serum and 0.1% triton X‐100. Sections were then washed three times with PBS and incubated for 4 h in goat anti‐rabbit IgG‐Alexa 488 (green; 1:500; Invitrogen) or goat anti‐rabbit IgG‐Alexa 594 (red; 1:500; Invitrogen) (Laboratories, Burlingame, CA) diluted in PBS supplemented with normal goat serum, and 0.1% Triton X‐100. Finally, sections were washed five times for 10 min in PBS, and cover‐slipped with Vectashield with DAPI mount (Vector; H-1500).

Immunofluorescent staining for the inflammatory markers of major histocompatibility complex (MHCII) and tumor necrosis factor α (TNF‐α) were conducted on every sixth coronal section throughout the entire SNpc and gut. In all animals, sections were anatomically matched. Slides of coronal sections (40 mm) from each rat were washed three times in 0.1 M PBS. The slides were then incubated in blocking solution for 1 h using PBS supplemented with 10% normal horse serum and 0.1% Triton X‐100. Sections were then incubated overnight at 4 °C with mouse monoclonal MHC II (anti‐RT1B (OX‐6); NB100‐65541; Novus Biologicals, Centennial, CO) or rabbit polyclonal TNF‐α (ab6671; Abcam, Cambridge, MA) antibodies in PBS supplemented with 10% normal horse serum and 0.1% triton X‐100. Sections were washed three times for 10 min in PBS and then incubated in PBS supplemented with 10% normal horse goat serum and 0.1% Triton X‐100 containing corresponding secondary antibodies, goat anti‐ mouse or rabbit IgG‐Alexa 488 (green; 1:500; Invitrogen), for 90 min. Finally, sections were washed five times for 10 min in PBS, and cover‐slipped with Vectashield with DAPI (Vector; H-1500). Sections were analyzed in independent channels with an Olympus FV1000 laser scanning confocal microscope equipped with Fluoview SV1000 imaging software. Control studies included exclusion of primary antibodies substituted with 3% normal horse serum in PBS. No immunoreactivity was observed in these controls.

To gain a general estimate of graft survival, alternate brain and gut tissue sections were processed for immunofluorescent staining with the human-specific nuclei marker (HuNu). Briefly, the mouse monoclonal antibody (HuNu, 1:100, Cat. No. 1281, Millipore Sigma, USA) was combined with the secondary antibody, monovalent goat anti-mouse Fab’ fragment conjugated to rhodamine (1:200; Cat. No. 115–607-003, Jackson ImmunoResearch, West Grove, PA), and incubated at RT for 2 h, followed by incubation in 1% normal human serum and 0.5% Triton X 100 in PBS for 30 min at RT and subsequently incubated with the previously prepared antibody cocktail overnight at 4 °C. The next day, the slides were thoroughly washed in PBS and coverslipped with Vectashield^®^ containing DAPI (Vector Laboratories, USA). The slides were then examined under epifluorescence using an Olympus BX60 microscope. Cells immunopositive for HuNu were counted manually from the entire slide at 40X and determined as a percentage of total nucleated cells for each mouse, providing a general estimate of engrafted hUCB cells.

### Parallel cell-based GBA mechanistic studies

In an effort to understand the direct role played by the gut on dopaminergic neurodegeneration, we then cultured dopaminergic cell line human dopaminergic SH-SY5Y cells (American Type Culture Collection, Manassas, VA) which were then exposed to homogenized gut from Tg or Wt animals. SH-SY5Y cells were cultured at a density of 1 × 10^4^ cells per well using 8-well poly-lysine plates (Nalgene Nunc, Rochester, NY) in DMEM (Invitrogen) with high glucose supplemented with 10% fetal bovine serum (FBS) (Invitrogen), 100 I.U/ml penicillin, and 100 μg/ml streptomycin (Mediatech, Herndon, VA). At 2 d after culture, SH-SY5Y cells were exposed to 1 μg of gut homogenate (including duodenum, jejunum, and ileum) from either Tg gut, Tg gut+P, Tg gut+hUCB+P or Wt gut for 24 h at 37 °C. Tissue homogenates were prepared in RIPA buffer containing protease inhibitor cocktail (ThermoFisher, Pittsburgh, PA) and diluted into PBS. The homogenate in PBS was passage through a 100 μm mesh filter (Miltenyi Biotec, San Diego CA). The homogenate was placed on a polycarbonate cell culture insert with 1000 μm mesh (Miltenyi Biotec, San Diego, CA) immersed in the SH-SY5Y cell culture medium but prevented gut homogenates to traverse the insert, allowing us to conduct accurate assessment of the SH-SY5Y cells without contamination from the gut homogenates. After this exposure, the insert containing the homogenate was removed and the cultured SH-SY5Y cells were analyzed for cell viability via the MTT assay (Promega, Madison, WI) and inflammatory response via the TNF-α assay (Abcam, Watham, MA) at the manufacturer’s recommended dilutions. Each treatment condition was replicated three times using three different cell cultures.

### Statistical analysis

The data were evaluated statistically using one-way analysis of variance (ANOVA) or two-way ANOVA and subsequent post hoc Bonferroni’s test. Statistical significance was preset at *p* < 0.05 (GraphPad version 5.01). Based on our stroke modeling experience, we expected a 15% estimate of variation within each group. This variance was similar between groups that were statistically compared.

## Data Availability

All data reported in this study are available from the corresponding author on reasonable request. All original data and protocols are available from CVB upon reasonable request.
